# A training program to extend the reach of the deciphering developmental disorders in Africa (DDD-Africa) study

**DOI:** 10.3389/fgene.2025.1611047

**Published:** 2025-10-03

**Authors:** Aaliah Charles, Zané Lombard, Nadia Carstens, Zandisiwe Goliath, Aimé Lumaka, Prince Makay, Nadja Louw, Robyn Kerr, Daniesha Govender, Amanda Krause, Koenraad Devriendt

**Affiliations:** ^1^ Division of Human Genetics, National Health Laboratory Service and School of Pathology, Faculty of Health Sciences, The University of the Witwatersrand, Johannesburg, South Africa; ^2^ Department of Internal Medicine, School of Clinical Medicine, Faculty of Health Sciences, University of the Witwatersrand, Johannesburg, South Africa; ^3^ Genomics Platform, South African Medical Research Council, Cape Town, South Africa; ^4^ Center for Human Genetics, University of Kinshasa, Kinshasa, Democratic Republic of Congo; ^5^ Center for Human Genetics, University of Leuven and University Hospitals Leuven, Leuven, Belgium

**Keywords:** developmental disorders, intellectual disability, low and middle-income countries (LMIC), genetic testing, training program, exome sequencing, Africa, deciphering development disorders in Africa (DDD-Africa)

## Abstract

Developmental disorders (DD), including intellectual disability (ID) and birth defects, affect approximately 7% of individuals worldwide, contributing to high mortality and lifelong morbidity. These disorders impose significant financial and psychological burdens on affected families. Genetic causes are identified in over 40% of DD cases, but diagnostic challenges, lack of appropriate management and curative treatments, and limited knowledge of natural history complicate management. Genetic testing, such as exome sequencing, is the standard diagnostic approach in developed countries. However, access to genetics services in low- and middle-income countries remains limited. Key barriers include poor access to specialist services in general, limited infrastructure, insufficient expertise in medical genetics, and outdated medical training curricula. The Deciphering Developmental Disorders in Africa (DDD-Africa) international training program initiative aims to address some of these disparities by establishing a network of trained professionals across African countries. This network will drive genomic research by identifying patients with DD, assessing them appropriately to direct genomic testing, and providing support to affected families. The training program consists of three phases: (1) an online course covering training in current core medical genetics concepts, (2) a 2-week on-site practical training in Johannesburg in identification, clinical assessment, and variant interpretation of DD patients, and (3) a hands-on implementation of the complete diagnostic process with four families recruited at each team’s home institute. The program trains healthcare professionals consisting of a clinician and laboratory scientist together, emphasizing the need for collaboration and a comprehensive understanding of integrated genetic clinical assessment and laboratory diagnostics. Ultimately, the initiative seeks to enhance diagnostic capabilities and family support, fostering a strong pan-African network in the field of DD.

## 1 Introduction

Developmental disorders (DD) are disabilities presenting during childhood with a lifelong impact. DD encompasses a spectrum of phenotypes, of which developmental delay or intellectual disability and birth defects (congenital anomalies) are the most common. The incidence of DDs varies geographically (Olusanya et al., 2022), and they collectively affect approximately 7.1% of individuals globally (Olusanya et al., 2023). Worldwide an estimated 240,000 newborns die annually within 28 days of birth from congenital disorders (World Health Organization, 2023). Importantly, 94% of children born with serious congenital disorders are in low- and middle-income countries (LMIC) (World Health Organization, 2023). In LMICs, advances in basic health conditions are reducing the burden of infections, making the impact of DD and birth defects due to genetic causes relatively more prominent (World Health Organization, 2023; Liu et al., 2015). A child with a DD results in a substantial financial burden for both the family and society (Genereaux et al., 2015). These conditions often result in lifelong disabilities requiring costly multidisciplinary care, such as surgery, physical therapy, speech therapy, special schooling and wider impacts of multiple hospital and clinic visits.

Alongside the financial and psychological burden, individuals with DD in LMIC face high levels of stigmatization and exclusion, with girls and women being disproportionately affected (Fairchild, 2002). Cultural and religious beliefs often contribute to the prejudice, with prevailing mystical or traditional beliefs on the causes of DD potentially hindering scientific progress (Mkabile et al., 2021; Scior et al., 2015). Such beliefs perpetuate a cycle of misinformation. These interconnected challenges highlight the urgent need for comprehensive support and action in addressing DD and birth defects in LMICs. In response, the World Health Organization has emphasized the need to develop expertise and build capacity to prevent birth defects as well as care for children with them (World Health Organization, 2010). The World Health Organization (2010) has also called for strengthened research on etiology, diagnosis, prevention of major birth defects, and the development of greater international cooperation to address these challenges.

The etiology of DD is highly heterogeneous, with genetic causes thought to be implicated in over 40% of cases (Shchubelka et al., 2024; Wright et al., 2023). One important aspect of care in DD is a timely and accurate etiological diagnosis. Reaching such a genetic diagnosis has implications beyond medical treatment and is considered a priority for patient care. Although a specific diagnosis rarely leads to curative treatment, it can direct management, enabling the child to receive essential services, and informing the family about the prognosis. Furthermore, it can inform the family about recurrence risks and provide accurate information for reproductive planning. In most cases, parents face the painful reality that despite their best efforts, the medical condition of their child cannot be cured. In other cases, due to the lack of curative treatment, parents are often disappointed and embark on a new journey of acceptance, grieving the loss of hope of having a healthy child. Reaching a genetic diagnosis also significantly increases parental quality of life, due to emotional relief and refutes feelings of guilt and self-reproach for the child’s disorder (Lingen et al., 2016). At a societal level, data on the etiology of DD provide a solid basis for disseminating correct information and changing false beliefs that harm children and their families, thus reducing discrimination and inequality. Scientific documentation of DD provides a basis to convince healthcare systems to consider hidden burdens and the need for care associated with a lifelong disability.

Genetic insights have the ability to fundamentally change the practice of medicine. The technological revolution, including Next-Generation Sequencing (NGS), has transformed our understanding of genomic variation and its relevance to health and disease. This has significantly impacted the precision of diagnosis for DD. There has been a rapid increase in knowledge of the genes implicated in DD, with 1483 definitive genes and 726 genes with strong evidence now documented (Gene2Phenotype, 2025). NGS techniques such as Whole Exome Sequencing (WES) have enabled testing of the whole coding genome simultaneously, rapidly increasing diagnostic utility. The overall cost is less than that of the cascade of conventional testing typically required to elucidate the genetic cause of a DD (Wiener et al., 2023). Despite the strong recommendations by professional societies to consider exome or genome sequencing as a first or second-tier test for patients with a DD (Manickam et al., 2021), access to clinical genetic services in most African countries is often absent or insufficient (Kamp et al., 2021). Deficiencies at several levels explain this shortcoming. In most African countries, knowledge and expertise in medical genetics is limited, since medical curricula fail to teach basic concepts of contemporary genetics and genomics and do not provide training for laboratory and clinical geneticists. As a result, no initiatives are taken to build the infrastructure for genomic testing. This is further hampered by the low resources of many health systems. Consequently, genetics is not integrated into medical practice, and patients’ needs are inadequately addressed.

While strides are being made to strengthen cross-continental training in genetics and genomics by the H3Africa consortium (Lumaka et al., 2022) and other initiatives like the African Society of Human Genetics (AfSHG), genetics expertise remains limited in many regions. These consortia have made significant efforts to build capacity, foster collaborations, and enhance training opportunities, which are gradually expanding the field across Africa (Munung et al., 2018; Mulder et al., 2018; Nembaware et al., 2023). There is a paucity of clinical geneticists and translation of training to patients. Furthermore, the overall limited expertise in genetics contributes to reduced participation of African teams in global genetics research. While the genetics research community has a strong tradition of collaboration and networking, African teams face barriers to accessing these networks due to insufficient expertise and a lack of personal contacts, which often serve as entry points. Clinical geneticists, who often deal with ultra-rare disorders, need networks to share clinical information, in order to improve patient management and care.

Introducing genomics in DD research in Africa requires addressing several local factors, including legal aspects (e.g., ownership and protection of research data), ethical concerns (e.g., informed consent, confidentiality, returning results to patients), administrative challenges (e.g., sample shipment), and cultural beliefs (e.g., views on the etiology of disabilities). In resource-poor settings, where unequal access to healthcare is a significant challenge, it is essential to ensure that diagnoses are made cost-effectively. As a first, and the most important, step to overcome individual and institutional shortcomings in genomics in the field of DD in Africa, we initiated a training project with several African countries. The primary aim of this training program is to implement the sustainable use of clinical genetic assessment and genomic testing as tools in the assessment, management and care of individuals with a DD and their families across the continent. In addition to strengthening diagnostic and technical capacities, the training program is designed to: (1) Foster the professional development of participants as future academic leaders through continued mentorship from experienced genetic and genomic specialists; (2) Support participants in contributing to curriculum development and mentorship within their home institutions by providing long-term access to course resources and encouraging the adaptation of training materials for local use; and (3) Promote sustainability through a training-of-trainers model, the formation of peer networks for mutual support and skill enhancement, and the strategic recruitment of participants with stable institutional affiliations to embed genomics capacity within existing healthcare and academic systems.

Through this initiative, we also aim to identify and address critical barriers (organizational, clinical and laboratory) to implementing genomics in DD in Africa. Our experience with DDD-Africa demonstrates that building a network provides crucial support for ongoing efforts, which greatly enhances long-term sustainability.

## 2 Pedagogical frameworks

**FIGURE 1:**
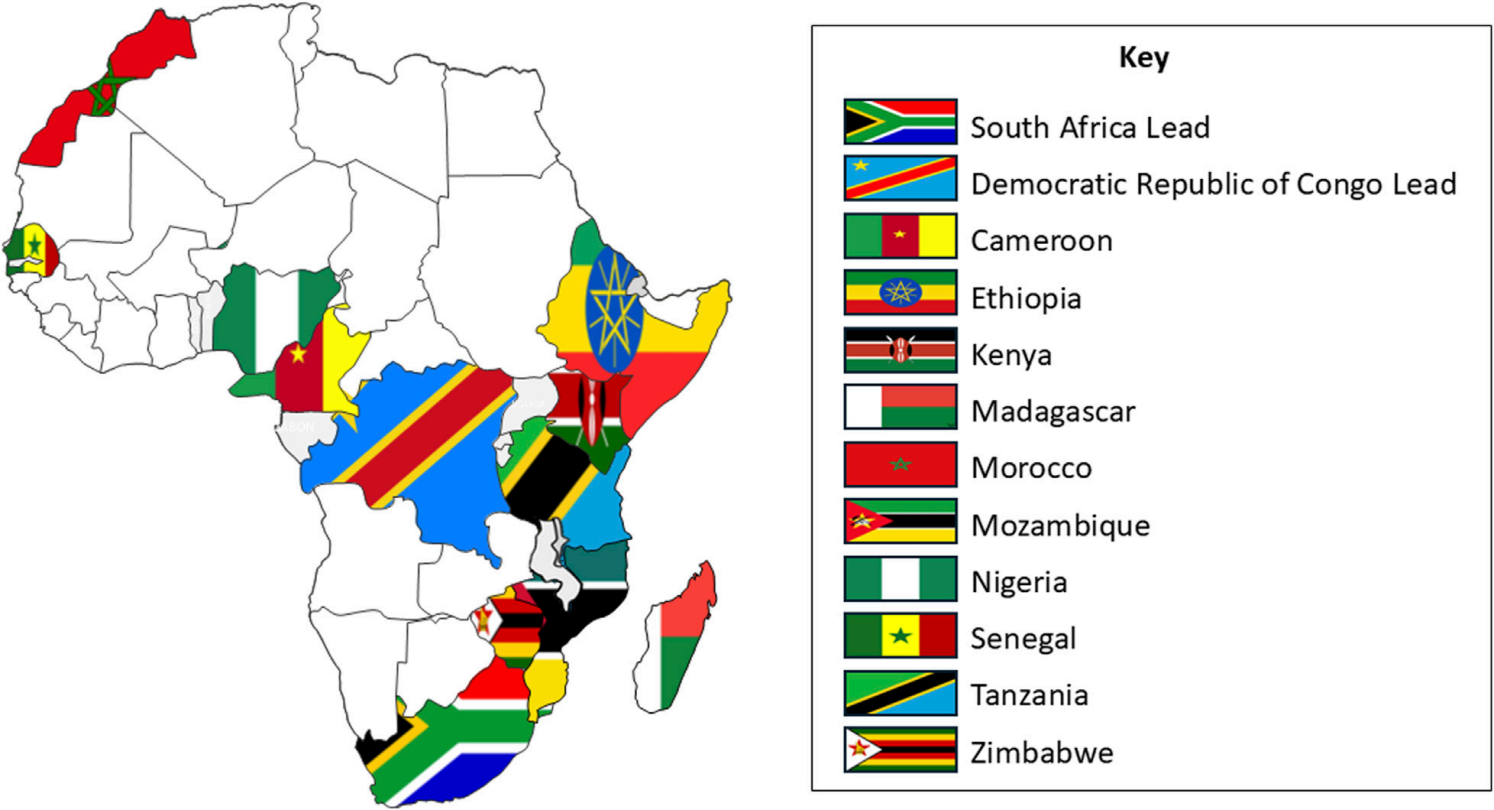
Teams selected to participate in the DDD-Africa training program are shown along with the South African and the Democratic Republic of Congo drivers of the training program.

The project called “Training teams to decipher developmental disorders in Africa” or “DDD-Africa training program” for short, received financial support from VLIR-UOS (grant number GO2023ITP249), the Flemish rectors’ conference in Flanders, Belgium (VLIR-UOS Webpage). Deciphering Developmental Disorders in Africa (DDD-Africa), a study funded primarily by the National Institutes of Health (NIH), served as a model and starting point (DDD-Africa (H3Africa) Webpage and DDD-Africa (Wits Human Genetics) Webpage). DDD-Africa aims to develop a sustainable genetic diagnostic platform for patients with DD in Africa. Drivers of this project are two academic centers: (1) The University of the Witwatersrand in Johannesburg, South Africa and (2) the University of Kinshasa in the Democratic Republic of Congo (Figure 1), whose previous collaborations, personnel and infrastructure capacities justify their selection as academic sites. This is supported by strong partners at the Wellcome Sanger Institute (UK) and the Center for Human Genetics of KU Leuven (Belgium).

### 2.1 Candidate selection for the training program

In LMICs, the current NGS infrastructure, where it exists, is geared towards infectious disease surveillance, with human genetic expertise lacking. The primary barrier to the genetic diagnostic process of DD is the requirement of a multidisciplinary team of trained clinicians and scientists with an integrated knowledge of human genetics and genomics. Clinicians must be trained in accurate and detailed clinical phenotyping, with a deep understanding of DD phenotypes and their variability, as these are crucial for patient selection and variant-guided phenotyping. Clinicians also play a vital role in shortlisting genetic variants by incorporating clinical information into the process, which directs the variant interpretation and informs further investigation and management. This “bedside to bench and back to bedside” approach ensures that clinical data continuously informs the diagnostic and treatment decisions. Furthermore, clinicians must provide ongoing psychosocial support to families throughout the diagnostic odyssey especially when no diagnosis is initially found—and continue to support them post-diagnosis as they cope with the implications of the findings.

Laboratory scientists play a crucial role in ensuring that samples are correctly obtained and processed. Additionally, they require the necessary skills to apply up-to-date bioinformatics tools to analyse sequencing data. Genetic variants must be analyzed and interpreted by this multidisciplinary team and fed back to the patient appropriately in their setting. Close and continued interaction between the clinician and the laboratory scientist is a necessity for success, as this ongoing interaction is key to effective patient diagnosis and care. For these reasons, we selected teams of duos consisting of a medical doctor and a laboratory geneticist, both from the same country and employed at the same or a nearby center. This composition was designed to facilitate a foundational genetic testing workflow, from patient phenotyping and sample collection to DNA extraction within the partner countries. The subsequent bioinformatic analysis needed will be through the core facility, supporting teams with access to trained bioinformaticians. This was a strategic decision that ensures access to high-level expertise despite budget constraints and the present limited availability of clinical bioinformaticians across the continent. Candidates were required to demonstrate a good level of insight and expertise in the field and have the ability to understand and communicate in English, as it was the official language of the course. To enhance sustainability, it was also a requirement for candidates to have a long-term appointment at an institution in one of the VLIR-UOS listed countries (https://www.vliruos.be/get-funded/calls/international-training-programme-2024) and willingness and motivation to act as drivers of change in their respective countries.

The training project was widely advertised and received 25 applications from 14 countries. The relatively low applicant pool was anticipated, as the program’s eligibility was restricted to specific countries by the funding agency. Furthermore, the requirement for a clinician-laboratory scientist duo and the current scarcity of clinical genetics experience in many African nations naturally limited the number of eligible teams, ensuring we selected pioneers in the field. After a stringent application, screening, and interview process, 10 African teams, from Cameroon, Ethiopia, Kenya, Madagascar, Morocco, Mozambique, Nigeria, Senegal, Tanzania and Zimbabwe, were selected (Figure 1).

### 2.2 DDD- Africa training curriculum development

**FIGURE 2:**
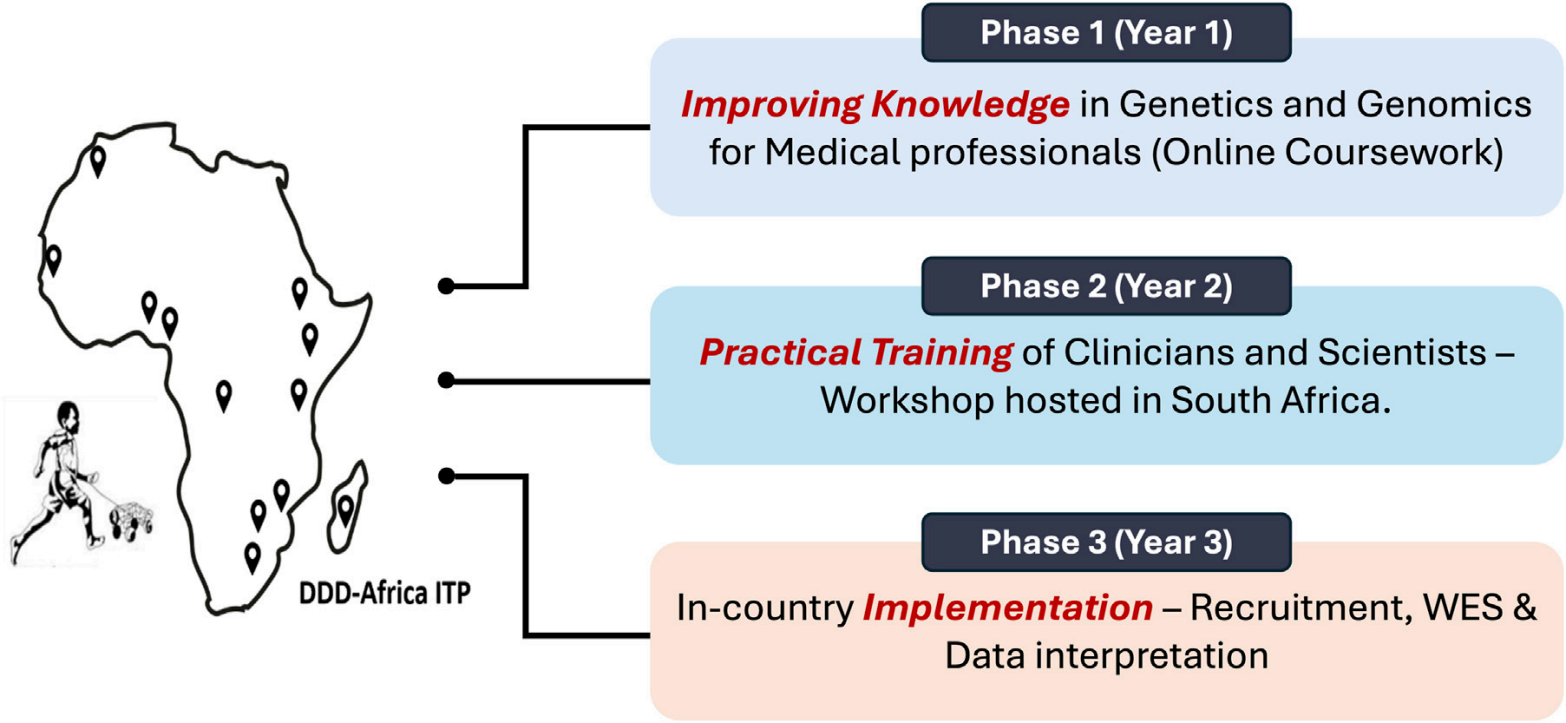
Objectives of the “Teaching Teams to Decipher Development Disorders” International Training Program (ITP). This interdisciplinary training program plans to meet the objectives in 3 consecutive phases as per the projected timeline. Abbreviations: DDD-Africa, Deciphering Developmental Disorders in Africa; ITP, International Training Program; WES, Whole Exome Sequencing.

To achieve our goal of training 20 healthcare professionals (10 clinician-laboratory scientist duos) from across Africa in utilizing genomic sequencing for the diagnosis, management and care of individuals with DD, we developed an innovative and multidisciplinary training program. This program is structured in three consecutive phases, each carefully designed to align with the objectives outlined in Figure 2. This design ensures a comprehensive and effective learning experience, enabling healthcare professionals to gain sufficient competency in medical genetics and genomics along with developing sustainable local expertise.

This program actively incorporates a combination of established models of adult and faculty education to optimize learning. Given that participants are full-time healthcare professionals, the curriculum combines synchronous (real-time) and asynchronous (self-paced) learning methods. Pre-recorded lectures allow participants to engage with the material at their own pace, while live discussions, hands-on laboratory work, and case-based clinical reviews promote active participation and skills development. Throughout phases 2 and 3, the training approach aligns with Kolb’s Experiential Learning Theory, cycling through concrete clinical and laboratory experiences, reflective observation, conceptual learning, and active experimentation (Wijnen-Meijer et al., 2022). These elements are especially reinforced during the implementation phase, where participants apply their knowledge in real-world settings within their home institutions. The design also reflects key principles from Knowles’ Adult Learning Theory, including self-directed learning, immediate applicability to clinical practice, and respect for the learners’ prior experience and professional roles (Bouchrika, 2024). The effectiveness of the training is being continuously evaluated using Kirkpatrick’s Four-Level Evaluation Model, assessing participant engagement, learning outcomes, changes in clinical behavior, and broader institutional or health system impacts over time (Rouse, 2011).

#### 2.2.1 PHASE 1: improving knowledge in genetics and genomics for healthcare professionals (2024)

During the first year of the training program, trainees participated in a structured online course designed to enhance their knowledge of medical genetics and genomics. The online course consisted of various didactic learning methods, including self-study with pre-recorded lectures, interactive online seminars conducted via an online communication platform, and reading research publications followed by online discussions. This blend of synchronous and asynchronous activities allowed participants to process the material in depth while continuing with their professional responsibilities. Expert trainers from the two partner sites in Africa (The University of the Witwatersrand in South Africa and The University of Kinshasa in the Democratic Republic of Congo), alongside those from KU Leuven in Belgium, facilitated the course. Existing courses at these partner sites were adapted to align with the program’s goals and the specific needs of Africa. The content was prepared to ensure relevance and practicality.

**FIGURE 3:**
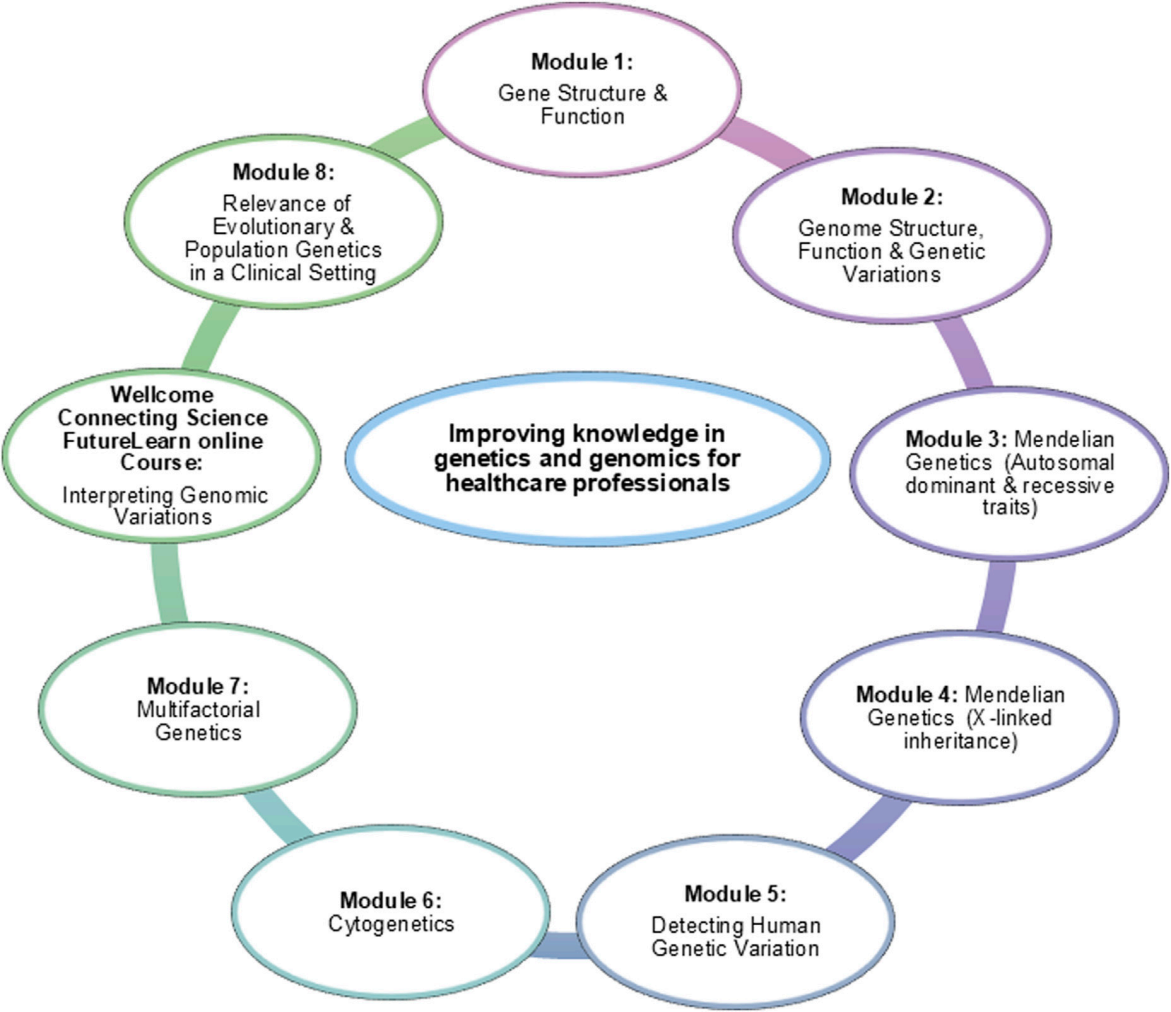
Topics of monthly modules completed during the first phase of the training program.

The course was divided into eight themed modules (Figure 3), with each module running over 4 weeks. Weeks 1 and 2 of the module focused on personal study, where trainees engaged with prescribed readings and pre-recorded lectures accompanied by lecture slides. The level of English proficiency varied among the trainees as three duos were from French-speaking countries, one from a Portuguese-speaking country and six from countries where English was spoken as a second language. The lecture slides were offered in both English and French to facilitate understanding of the topics. Week 3 involved a live seminar session, allowing participants to clarify concepts, engage in real-time discussions with experts, and apply their knowledge. In Week 4, an assessment was conducted to evaluate progress and engagement with the course content, and identify areas of difficulty. Feedback on the assessment was provided, and individual trainees requiring further support were given a remediation plan to ensure they stayed on track for successful course completion. Participants were also given the opportunity to provide anonymous feedback after each module through a survey. This survey included the evaluation of the clarity of the learning objectives (Supplementary Table 1), the appropriateness of the reading materials, the quality of the pre-recorded lectures, and the overall delivery of the module. Trainees also had the opportunity to suggest improvements or comment on their learning experience. This feedback mechanism ensured that the program continuously adapted to the needs of its participants, further supporting its long-term success.

The course materials, including recorded lectures and reading resources, were uploaded onto the University of the Witwatersrand Canvas learning management platform, locally branded as” ULWAZI” (Canvas LMS link). These materials were developed in line with Creative Commons copyright licenses to ensure wide sharing and sustainability of the training program (https://creativecommons.org/).

Additionally, to the eight themed modules, trainees completed the Wellcome Connecting Science course entitled, “Interpreting Genomic Variation: Overcoming Challenges in Diverse Populations”, which is hosted on the FutureLearn online learning platform (FutureLearn Course: Interpreting Genomic Variation). This 3-week course guides learners through the process of variant classification and interpretation, contextualized for diverse populations. Three co-investigators of the DDD-Africa project (Profs Zané Lombard, Amanda Krause and Aimé Lumaka) were developers and educators on this course. This course also served as the foundation for the Phase 2 variant analysis sessions.

#### 2.2.2 PHASE 2: practical training of clinicians and laboratory geneticists (January 2025)

Phase 2 of the training program was hosted by the Division of Human Genetics, at the University of the Witwatersrand and the National Health Laboratory Service (NHLS) in Johannesburg, South Africa. The selected 10 duos were invited to attend this on-site training workshop in South Africa. This phase took place in January 2025 over a period of 15 days, with 10 days of intensive training. It focused on practical, hands-on training within a context reflective of African infrastructure and patient populations. Trainers from KU Leuven (K.D.), the University of the Witwatersrand (AK., Z. L, N.C), and the University of Kinshasa (A.L, P.M) were invited to take the lead on various topics based on specialized expertise.

We opted for a joint program for the clinicians and laboratory scientists, with a good balance of clinical and laboratory aspects of DD. This interdisciplinary approach aimed to foster collaborations within the duos and ensure all participants developed a shared understanding of the entire diagnostic process.

After an introductory lecture on etiological and diagnostic aspects of DD, the course followed key steps of the medical genetic diagnostic workflow–from consultation and clinical assessment to genetic testing, interpretations and returning of results. The importance of genetic counseling was also emphasized (Supplementary Table 2). Anonymized patient cases (with a genetic diagnosis from patients who were recruited earlier in the DDD-Africa project) were assigned to each team and followed throughout the course. This anchored the learning experience in real-world clinical challenges and helped consolidate the knowledge across diagnostic pathways. Bioethical considerations and broader ethical, legal and societal implications (ELSI) relevant to genetic testing in Africa were discussed, including issues such as data protection and consent procedures. Various methods, including lectures, pre-recorded videos, role play, laboratory demonstrations, hands-on practice and bioinformatic sessions, were used to engage participants in the learning process. Each module was structured with theory and practice components, ensuring that trainees gained both theoretical knowledge and practical skills.

#### 2.2.3 PHASE 3: implementation phase (February 2025- September 2026)

**FIGURE 4:**
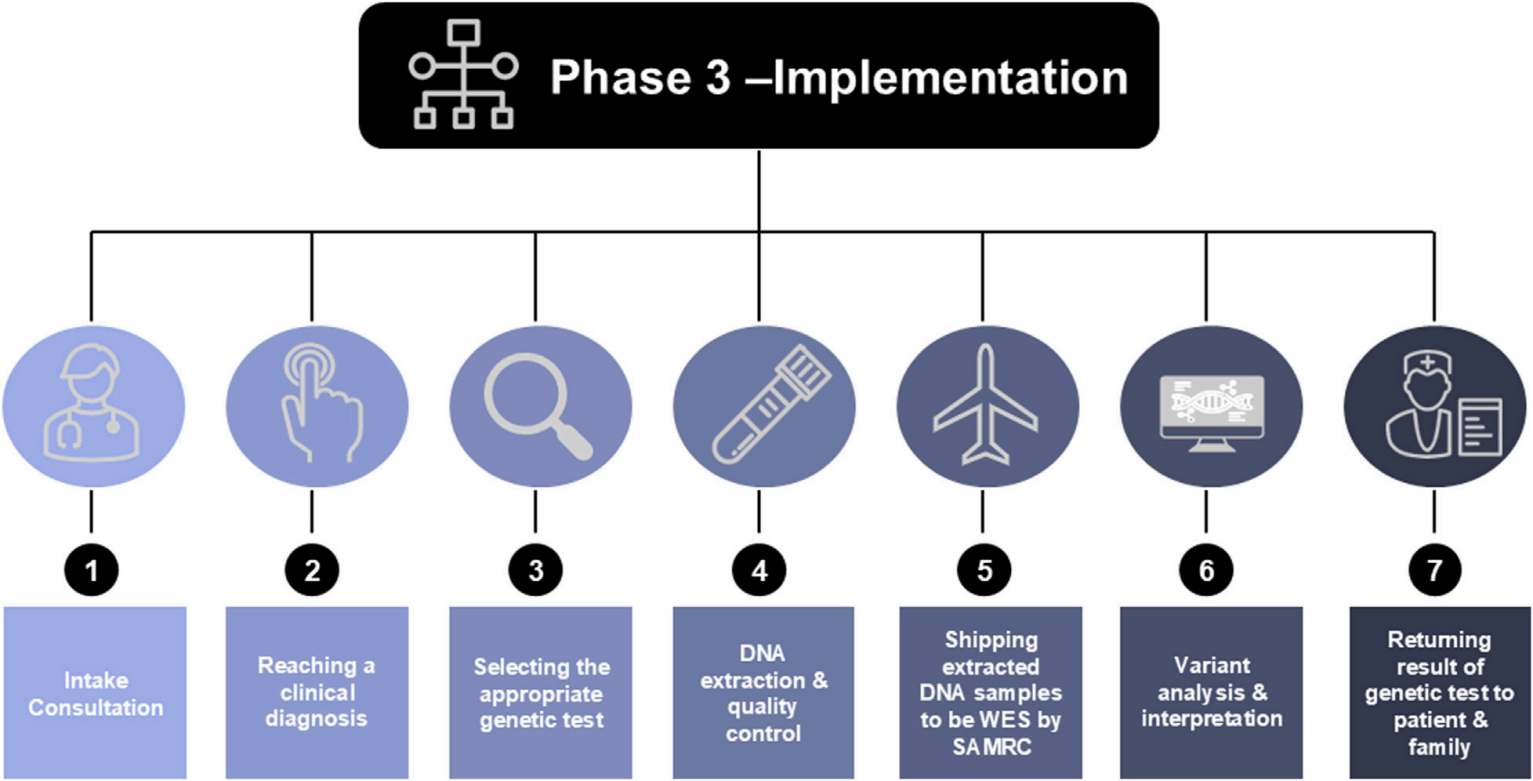
The implementation process of phase 3, from patient consultation to returning genetic results to the patient and their families. Abbreviations: WES, Whole Exome Sequence; SAMRC, South African Medical Research Center.

During Phase 3, the focus will shift from theoretical knowledge to practical application, where trainees will implement the diagnostic process from patient intake consultation to the delivery of genetic testing results (Figure 4). Each team will recruit four trio families (a child with DD and their parents), who will undergo a multi-disciplinary clinical review followed by WES, in collaboration with the South African Medical Research Council (SAMRC) Genomics Platform. Due to the expensive nature of WES machinery, the in-country implementation phase relies heavily on shipping samples to central sites, which does not allow for full implementation locally. However, trainees will be supported while they navigate their institutional Review Board (IRB), local ethics approval processes, and implement each step of the diagnostic workflow. Key aspects of this phase include ongoing support meetings, transportation of DNA samples, and the secure storage and sharing of clinical information.

##### 2.2.3.1 Support during phase 3

Trainees will be supported through online engagements, serving different purposes: (1) Administrative meetings, where obstacles regarding ethical approval, MTAs, export and import permits, DNA shipment, transfer of sequence results, etc. Will be discussed. (2) Clinical case review meetings with discussions on clinical data from candidate patients for recruitment and discussions on identified variants and their classifications in later stages. Identified variants will also be evaluated considering the clinical information, with suggested implications for patient management based on the results. To increase the efficiency of such meetings, the data will first be evaluated in so-called internal meetings, with the local team and representatives from one of the 3 organizing centers, followed by external review meetings (with all the participants and organizing centres). (3) Variant classification training: During the second phase, students acquired the basic skills in variant classification and interpretation. To maintain these skills, illustrative additional training cases will be provided for self-study, followed by online discussions. (4) Additional teaching sessions will be organized to complement the teaching during this phase. Topics will include splicing, genetic mosaicism, copy number variant interpretation, epigenetics, application of FACE2GENE, and the use of alternative open-source diagnostic tools, like SEQR (Pais et al., 2022), for variant filtering. An important component of this ongoing training will be an online lecture dedicated to the complex ethical and practical considerations of incidental findings. The development of protocols for returning or not returning, such results will be guided by this training and tailored to the specific regulations and healthcare contexts of each partner country.

##### 2.2.3.2 Obtaining blood samples and transport of DNA

Obtaining biological samples (blood and DNA) and performing genetic analyses on the index patient and their parents requires preliminary approval from the local ethical committee, as well as informed consent from all participants. Each team was trained on DNA extraction and quality control to build local capacity. Good quality DNA samples are important to the success of any NGS-based testing. The DDD-Africa trainers and mentors will work with each team individually to ship the DNA samples to the sequencing facility in a practical way that would conform to the ethical and legal regulations of their country. This will include Memorandums of Understanding (MoUs), Material Transfer Agreements (MTAs) and permits as required.

##### 2.2.3.3 Storage of patient information and images

Clinical information (such as pedigree data, clinical notes, and photographs which for which specific informed consent was obtained following established guidelines; [Dysmorphology Subcommittee of the Clinical Practice Committee, 2000]) will be treated with strict confidentiality and pseudonymized using a unique coding system indicating the country of origin, the family, the relationship of family members to the index patient, and the affected individuals. For the purpose of the training, data (with explicit consent from the family) will be shared with other participating teams for training and research purposes. In line with the DDD-Africa program, data sharing will be facilitated through three separate databases, each serving a distinct purpose. (1) The DDD-Africa Research Electronic Data Capture (REDCap) database. Each duo will collect and manage study data using REDCap electronic data capture tools. REDCap is a secure, web-based software platform designed to support data capture for research studies (Harris et al., 2019; 2009). To aid uniformity, clinical information will be captured on REDCap using Human Phenotype Ontology and a standardized clinical data sheet. Consent forms and other documents will be scanned and uploaded onto the REDCap database. (2) DECIPHER (Database of Chromosomal Imbalance and Phenotype in Humans using Ensembl Resources) is an interactive web-based database, which supports genetic variant interpretation by means of a wide array of up-to-date tools (Firth et al., 2009). This will serve as a repository for the genomic variants identified in the study population. (3) FACE2GENE (http://www.fdna.com/face2gene/) is a morphometric analytic tool that offers an objective evaluation of the facial *gestalt* from 2D facial images. It allows for the storage and sharing of clinical photographs and assesses the presence of facial dysmorphism in an objective way. It has the potential to contribute to a reliable syndrome diagnosis by matching the face of a patient with similar patients in a database of individuals with known syndromes.

## 3 Results and discussion

This section presents the key individual outcomes and institutional/social outcomes the DDD-Africa Training Program achieved, and aims to achieve, along with the development and sustainability of resources, and the establishment of collaborative networks. Furthermore, we discuss the data gathered or planned, and the practical implications and objectives of the program, as well as the lessons learned during the implementation of the first 2 phases.

### 3.1 Potential individual, institutional and social outcomes

**FIGURE 5:**
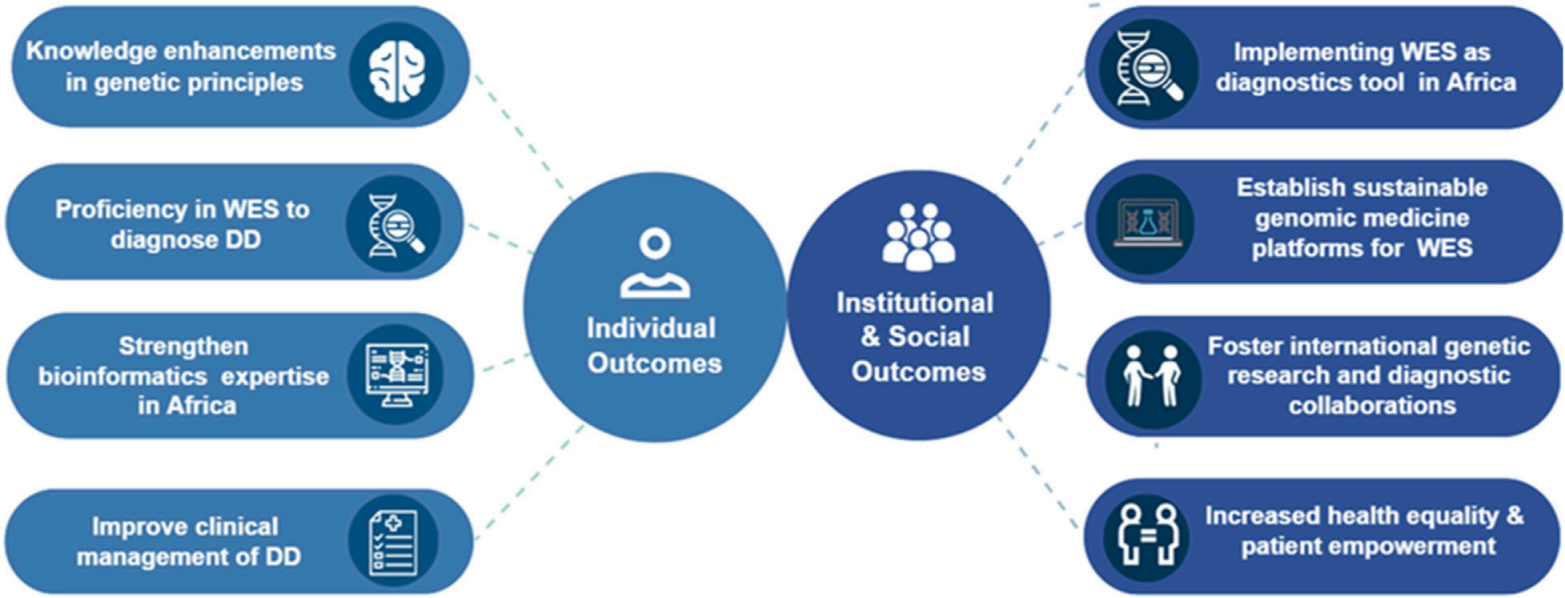
Potential individual, institutional and social outcome of the DDD-Africa training program.

The DDD-Africa Training Program has yielded several key individual, institutional and societal outcomes, contributing to the professional development of participants and setting the stage for further growth in subsequent phases (Figure 5). On an individual level, participants have enhanced their understanding of genetic principles during the first 2 phases, focusing on medical genetics and the application of genomic sequencing to diagnose DD. This newly acquired knowledge allows clinicians and laboratory scientists to make more informed and accurate diagnoses, directly impacting clinical outcomes. Another central achievement has been the enhancement of bioinformatics expertise in Africa. Participants have gained the tools and skills necessary to interpret genomic data effectively (phase 2), which will continue to be applied and expanded in their local institutions, improving genetic diagnostics. Through the integration of genomic data in clinical practice (phase 3), participants will be improving the clinical management of DD in their country. Another important outcome that will be achieved by the end of this training program is proficiency in using WES for diagnosing DD. Participants will learn how to successfully apply WES technology in their diagnostic work, which has the potential to improve both the speed and accuracy of diagnoses for DD in their local contexts. Throughout the course, the clinical-scientist teams have been encouraged to engage with their institutions and local support groups. The intention is that they will start to include other team members and will act as the centre in each country to start interacting with local stakeholders to improve genomics services and access.

The program is also anticipated to drive significant institutional and social outcomes, laying the groundwork for the sustainable integration of genomic medicine in Africa, as well as fostering collaboration on a global scale (Figure 5). The program has also worked to establish genomic medicine platforms in local institutions, ensuring that the gained skills for genetic testing and diagnostics are part of a sustainable model. These platforms will allow for long-term integration of WES and similar technologies into routine clinical practice. This process will contribute to improved personalized care for individuals with DD, which will be increasingly important as genomic diagnostics are implemented more widely. Through the program, international collaborations between African institutions and global research organizations have been fostered. These partnerships create opportunities for shared research, training, and access to expertise, which will continue to improve both research and diagnostic capabilities in Africa. As a result of the program, we anticipate a marked improvement in health equity and patient empowerment. With improved access to genetic diagnostics, patients will have a better understanding of their conditions and treatment options, contributing to more equitable healthcare outcomes.

### 3.2 Development and sustainability of resources

One of the key achievements of the program thus far has been the development and sustainability of the educational resources created throughout the training process. The online course materials, including pre-recorded lectures, reading resources, and assessment tools, have been designed to be scalable and adaptable for future trainees. These materials, hosted on the Canvas “ULWAZI” learning management system (LMS), remain accessible to all trainees, ensuring that participants can revisit and engage with the content even after phase 1 ends. ULWAZI has been instrumental in delivering the course content, tracking participant progress, and enabling seamless communication between trainers and trainees. The system supports both synchronous and asynchronous learning, allowing participants to engage with the material at their own pace while benefiting from live sessions and interactive feedback. These resources have been designed to meet not only the needs of the current cohort but also to serve as a foundation for future programs, contributing to the sustainability of the training. Additionally, the course materials were specifically adapted to address the unique healthcare settings in Africa, ensuring relevance across different regions. The availability of materials in both English and French enhances accessibility, allowing participants from diverse linguistic backgrounds to benefit equally. The sustainability of the training is further supported by the fact that the course materials can be continuously updated to reflect new developments in genomic research and local health challenges. The ULWAZI platform also allows for ongoing support and communication, with space for trainee interaction, feedback, and discussions on emerging challenges. This is seen as a key aspect of maintaining continuous learning after the formal training phases are completed.

The success of the DDD-Africa Training Program can also be attributed to the systematic processes and tools implemented throughout the course. These include the integration of bioinformatics platforms, and clinical case management tools to support both educational and practical training. Participants have been trained in using tools (such as DECIPHER and FACE2GENE), which are essential for interpreting genomic data. DECIPHER is primarily used for storing clinical and variant data, allowing for some level of variant interpretation and comparison with other patients in the database. FACE2GENE, on the other hand, compares vectorized photos to support phenotypic analysis, though it is limited to a small number of diseases and has shown limited performance in Africa (Bresler, 2024). These tools, despite their limitations, support the accurate analysis of genetic variants and phenotypic features, aiding in the identification of DD-related mutations.

### 3.3 Establishment of networks and collaborative relationships

Another significant result of the program has been the establishment of networks and collaborative relationships among trainees, instructors, and institutions. Participants have built strong cross-continental professional relationships that will hopefully extend beyond the duration of the training program. Through collaborative learning, joint case studies, and shared resources, a network of clinicians, laboratory scientists, and bioinformaticians has been committed to improving the diagnostic landscape for DD in Africa. The training has also strengthened institutional collaborations, with various African universities and medical centers, as well as international partners, such as KU Leuven in Belgium, involved in the program. These partnerships are expected to continue post-training, fostering the exchange of knowledge, resources, and technical expertise in genomics and medical genetics. The onsite training program in phase 2 has also allowed trainees to foster informal peer-support networks where they can share challenges and seek advice on cases that arise in their home institutions. The existence of these networks suggests that the program has laid the groundwork for a lasting community of practice in genomic medicine across Africa.

### 3.4 Qualitative feedback and insights from trainers and participants

Throughout the program, feedback was gathered both formally and informally from participants and trainers. Key themes that emerged include: (1) Appreciation for the interdisciplinary approach: both clinicians and laboratory scientists expressed high satisfaction with the integrated approach of the program. They found the balance between clinical knowledge and laboratory practices beneficial for fostering a more comprehensive understanding of DD diagnostics. (2) Challenges of contextual relevance: Some trainers noted that while the program’s content was relevant, there were ongoing challenges in fully adapting the training to the diverse contexts of the various participating institutions. This reflects the complexity of addressing different infrastructures, resources, and local health practices across countries. The diverse healthcare infrastructure and varying levels of resources across the participating African institutions pose challenges (e.g., not all countries involved have immediate access to DNA extraction equipment and laboratories) to the complete standardization of the training. However, the program’s ability to adapt and customize the learning experience for each context has been a strength.

### 3.5 Data gathered or planned, and outputs

Data collection is an ongoing process in the DDD-Africa Training Program, with both qualitative and quantitative data being gathered through various methods, including participant surveys, in-person interviews, and feedback forms. Although surveys for phase 2 have not been completed yet, we anticipate collecting feedback on the impact of the training program on participants’ clinical practice, bioinformatics capabilities, and knowledge of genetic principles. This data will provide valuable insights into the effectiveness of the program and areas for further improvement. Additionally, data collected from the ongoing implementation phase (phase 3) such as patient diagnoses, variant identification, and clinical outcomes—will also be collected. These data will not only inform the diagnostic accuracy of the trainees but also help track the long-term outcomes of implementing WES in African clinical settings. The outputs of this initiative are anticipated to extend beyond individual patient diagnoses to contribute significant operational and scientific insights. We expect subsequent publications to address the feasibility of local DNA extraction in resource-limited settings, the comparative landscape of ethical approvals and sample export permits across different African nations, and the aggregate genetic findings and rare disease case reports from the ten participating teams. These outputs will provide a valuable evidence base for the global genomics community and opportunities for scientific writing.

### 3.6 Lessons learned

Several important lessons learned have emerged throughout the course of the program, which will guide the future phases of the training: (1) Adapting to local contexts: Local infrastructure challenges, such as access to DNA extraction facilities and the shipment of biological samples, must be taken into account when designing future training programs. While this program is designed for in-country implementation of genetic testing, the sequencing phase is currently centralized at expert sites to ensure data quality and reliability, a critical factor for training efficacy. This approach acknowledges the present reality that clinical-grade exome sequencing infrastructure is not yet universally available across partner countries. In many of the countries, the extraction of high-quality DNA under diagnostic conditions is also not established. A key future goal will be to support the development of local laboratory structure and sequencing capabilities as technology becomes more accessible. (2) Flexibility in training delivery: The success of the blended learning model, which combines both asynchronous and synchronous learning, highlights the value of offering flexible training options. This allows participants to balance professional responsibilities with training while ensuring that they still receive comprehensive and hands-on learning experiences. (3) Building stronger support networks: There is a need for continuous support throughout the program. This includes regular follow-up, troubleshooting, and opportunities for further education when gaps in knowledge are identified, to ensure that participants continue to develop their skills and integrate genomic medicine into their practice.

This unique training project addresses a thus far unmet demand for integrated clinical and genetic diagnosis of individuals with DD. Now at the beginning of the third implementation phase, the first evaluation was positive. The trainees have demonstrated strong commitment to the program, and made remarkable progress, both with regard to the knowledge and insight into the basic principles of genetics in general, and the genetics of DD in particular. They have acquired the skills needed to start the third phase, i.e., from patient to diagnosis and back. The trainees have responded enthusiastically to the program. A major point contributing to its success is the unique composition of healthcare professionals. We train, in each institute, an interdisciplinary team consisting of both a clinician and a laboratory scientist. The training program was the same for both, reinforcing their function as a team and allowing them to get acquainted with the entire process of genetic diagnostics. Moreover, during phase 2, the teams were assigned a case study of an actual patient, and were required to follow the different steps required, leading to a genetic diagnosis. This contributed significantly to their learning curve. The initiative will create local nodes of knowledge and expertise in diverse African countries, which is important to address disparities in access to genomic medicine. We will strongly invest in the implementation phase, where the duo will build the infrastructure and apply their new skills in their local communities, in this way increasing sustainability. Sustainability and networking will be further promoted by linking the teams to the parent DDD-Africa study and to the international community of geneticists involved in DD, more specifically ITHACA, the European reference network for DD. We anticipate that these links may offer novel training and research opportunities. Ultimately, a strong pan-African network in the field of DD and rare diseases may contribute to defining priorities for the continent and setting the agenda for the coming decades.

This initiative aligns closely with global efforts to strengthen the health workforce and expand faculty training in genomics and rare diseases, particularly in under-resourced regions. By equipping interdisciplinary teams with both clinical and laboratory expertise, and by fostering long-term mentorship and institutional embedding, the program directly supports the WHO’s vision for capacity building in precision medicine. This program will contribute to a growing international movement to decentralize genomic medicine and empower local professionals to serve as educators, researchers, and leaders in their own contexts.

### 3.7 Future directions

Looking ahead, future iterations of the program will seek to deepen its educational and developmental dimensions by incorporating additional conceptual frameworks that further enrich trainees’ understanding of developmental disorders. These include greater emphasis on interdisciplinary knowledge-sharing beyond core genetics, the inclusion of exposome-related influences and neurodevelopmental trajectories in shaping health outcomes, and the integration of concepts such as neuroplasticity and diagnostic uncertainty. These elements will not only enhance the scientific rigor of the program but also strengthen the capacity of trainees to engage with complexity and ambiguity in real-world clinical settings. Furthermore, the program aims to more explicitly position participants as future educators, mentors, and research leaders within their institutions and regions. By evolving in these directions, the DDD-Africa program will continue to build a resilient, forward-thinking genomics workforce aligned with continental and global priorities in equitable healthcare and precision medicine.
