# Supplementary material for: A training program to extend the reach of the deciphering developmental disorders in Africa (DDD-Africa) study
Source: Front Genet. 2025 Oct 3;16:1611047. doi: 10.3389/fgene.2025.1611047 (PMC12532005; doi:10.3389/fgene.2025.1611047)
Supplement: Supplementary file 1 [file Table1.docx]

| **Table 1.** Overview of Monthly Online Course Topics and Learning Outcomes | |
| --- | --- |
| **Module** | **Key learning objective** |
| Gene structure and function | Describe the basic structure of DNA, defining 5’ and 3’ ends  Name and identify the parts of a typical human gene  Briefly explain the role of the promoter, enhancers, introns, exons, splice sites and the start and stop codons.  Define ‘mutation’ and describe the possible net effects of a mutation  Explain the difference between loss of function (LoF) and gain of function (GoF) mutations  Distinguish ‘inherited’ versus ‘de novo’ mutation  Summarize the different types of mutations (point / indels / splice) and discuss their possible impact on the protein  Illustrate how the position of a single nucleotide variant, with respect to a gene, has potentially very different consequences for the protein  Be able to read and interpret mutation nomenclature |
| Genome structure and function & human genetic variation | - Understand the concept of genetic variations and types of variations - Explain the origins of genetic variations - Use appropriate language to map genetic variation and to decode genetic results |
| Mendelian Genetics Part 1: Autosomal dominant and autosomal recessive | - Recognize pedigrees with a clear autosomal dominant (AD) or autosomal recessive (AR) disorder - Calculate recurrence risks for relatives of a person with an AD or AR disorder - Explain the difference between penetrance & variable expression - Recognize genetic conditions where de novo mutations are frequent - Distinguish the different types of mosaicism - Calculate the risk for relatives of a persons with an AR disorder in a family with consanguinity - Recognize the possibility of uniparental disomy in person with an AR condition - Recognize the occurrence in a pedigree of pseudo-dominance, semidominant or carrier manifestations - Able to look up whether a specific condition is AD or AR |
| Mendelian Genetics Part 2:  X-linked inheritance | - Explain the sex ratio at different ages - Pedigrees with a clear X-linked recessive or X-linked dominant inheritance - Calculate recurrence risks for relatives of a person with an X-linked recessive or dominant disorder - Identify obligate female carriers for an X-linked recessive disorder in a family - Explain why X-linked intellectual disability is as common in males as in females - Identify different ways carrier females for an X-linked disorder may have manifestations. - Explain why Turner syndrome is associated with manifestations - Understand the consequences of pathogenic variants of the different Y-linked genes |
| Detecting human genetic variation | - Improve the ability to choose the right molecular test - Acquire a better understanding of the test process for different genetic tests |
| Cytogenetics | - Describe the basic structure of a chromosome, including the types of chromosomes - Describe the normal human chromosome complement - Distinguish between types of numerical chromosome abnormalities - Explain the mechanisms by which numerical chromosome abnormalities arise - Outline some of the clinical features and complications of common aneuploidy syndromes - Describe different types of structural chromosome abnormalities - Outline some of the clinical features of a common microdeletion syndrome - Briefly describe the different techniques used to analyze chromosomes: Karyotype, FISH, QF-PCR, Chromosomal microarray - List the advantages and disadvantages of each technique - Identify which test would be most appropriate to use in different clinical scenarios |
| Multifactorial genetics | - Recognized that both genetics and the environment play a role in multifactorial traits and -diseases. - Describe how we detect genetic contributions to multifactorial traits. - Indicate whether population differences exist in susceptibility for multifactorial diseases. - Appreciate the clinical utility of understanding the genetics of multifactorial diseases. - Outline and describe the study design considerations for multifactorial disease studies - Explain why careful definition of phenotypes is important in genetic studies of multifactorial diseases. - Explain what population stratification is and evaluate how this can influence an association study’s outcome. - Explain how GWAS data is visualized. |
| Wellcome Connecting Science FutureLearn Course: Interpreting genomic variation- Overcoming challenges in diverse populations | - Use tools for genomic variant classification and interpretation in different contexts - Interpret and apply international guidelines for variant classification and interpretation and dealing with uncertainty - Explain how changes in the understanding of biological implications of variants affect their classification and interpretation - Assess the implications of clinical context in dealing with variants of unknown significance - Recognize the challenges in variant classification and interpretation in diverse populations and resource-limited settings |
| The relevance of population and evolutionary genetics in a clinical setting | - Describe why the evolutionary history of the emergence of our species (anatomically modern humans, homo sapiens) is relevant to clinical genetics - List and describe the evolutionary processes that have shaped genetic diversity - Explain the concepts of bottlenecks and founder effects and how they are recognized and relevant to a clinical setting - Give examples of natural selection and its effect on allele frequencies - Understand the effect of consanguinity on population prevalence of autosomal recessive disorders - Define and understand the implications of allelic heterogeneity and locus heterogeneity in the context of monogenic disorders - Distinguish the high-level differences in the genomes of African populations compared to non-African populations - Describe why size does matter in genome-wide association studies for complex multifactorial diseases and traits - Understand the limitations of the transferability of polygenic scores (polygenic risk scores) between different populations |

**Table 2.** Topics covered during phase 2 - Onsite training at NHLS and the University of the Witwatersrand, Johannesburg.

| **Topic** | **Aim** | **Theory** | **Practice** | **Formal documents and Materials:** |
| --- | --- | --- | --- | --- |
| Intake Consultation | To learn to collect comprehensive clinical information during the consultation of a patient with a DD (and their parent(s). This includes personal and family history, clinical examination (with special attention to congenital anomalies and dysmorphism), as well as engaging with the complex psychosocial issues around a child with DD. | Interactive Lecture | Role play (in two groups of 5 teams each). Trainers play the parents of the case assigned to each team. Team members act as clinicians and receive the parents at the consultation. They were supported by experienced genetic counsellors during this consultation. Clinical pictures of the patient were shown. | - PowerPoint presentation: the intake consultation - PowerPoint presentation: how to take clinical pictures - Clinical information collection sheets - Background articles: including pedigree symbols, elements of morphology (article series AJMG) |
| Reaching a clinical diagnosis | To learn the principles of grouping the different clinical elements into an overarching diagnosis or diagnostic category. | Lecture | Each team presents the summary of the clinical data of their case, as collected during the role play. This includes an evaluation and integration of the dysmorphological aspects, together with the history and examination, based on the clinical pictures, and trying to reach a short differential diagnosis. There follows a group discussion with all trainees. | - PowerPoint presentation: reaching a clinical diagnosis - Background article: Hennekam RC et al., 2013. Elements of morphology: General - Terms for congenital anomalies. Am J Med Genet Part A 161A:2726–2733. - Gorlin slide collection |
| Selecting the appropriate genetic test | To understand the different routine genetic laboratory tests available for the diagnosis of common genetic disorders and to learn how to select the most appropriate test depending on the suspected diagnosis. | Lecture | Laboratory tour, where different techniques are explained. A printout of 5 tests is given, and students are asked to match these with one of five common and illustrative genetic disorders: QF-PCR (Down syndrome), Sanger sequencing (Neurofibromatosis type 1), NGS (Neurofibromatosis type 1), chromosomal microarray CGH (22q11.2 deletion syndrome and Wolf-Hirschhorn syndrome caused by an unbalanced translocation), fragment analysis (Fragile-X syndrome). This is followed by a group discussion. Key aspects of a laboratory report are illustrated for the five disorders. | - PowerPoint presentation: different genetic laboratory tests - For 5 disorders: clinical vignette, printout of laboratory result, laboratory report. |
| DNA extraction & quality control | To learn the theoretical basis of DNA extraction, quality control and storage, and train in the laboratory to extract DNA using a kit (FlexiGene DNA kit from Qiagen). This kit will be provided to extract DNA in their home institutes for patients to be included in this training program. | Lecture & video showing the process of DNA extraction | The students practiced DNA extraction in the laboratory, using the Flexigene DNA extraction kit. The concentration measurement using nanodrop and quality control (by means of gel electrophoresis) were demonstrated. | - PowerPoint presentation on DNA extraction and quality control - Video on DNA extraction - Protocol for DNA extraction using the kit |
| Variant analysis & interpretation | Students will learn the different steps of variant classification and interpretation, starting from the variant calling (VCF)-files. | Lectures on the medical and psychosocial aspects of a genetic diagnosis | The students will practice in the computer class the process of variant classification in two phases:   - The clinical phenotype of a series of 5 test cases with a known genetic developmental disorder are shown. For each of these, the students are given the pathogenic variant(s) identified, and they learn in the computer class how to collect and evaluate the different parameters that can be used to classify this variant. - Each team will then obtain, for their assigned clinical case, a series of variants (SNV’s) identified. They will classify each of these variants with the aim of identifying the (likely) pathogenic variant.   Additional cases were available for analysis for teams making rapid progress. | - PowerPoint presentations: introduction to variant filtering and curation; filtering demo (from excel to DECIPHER). - Background articles - Online video introduction on DECIPHER: <https://www.youtube.com/watch?v=kfYwt52wJws> - Video: course on variant classification and interpretation. - ACMG-AMP Guidelines paper: <https://pubmed.ncbi.nlm.nih.gov/25741868/> |
| Returning results of genetic testing to the patient and family | To learn how to evaluate the clinical significance of an identified pathogenic variant and return this information in a clinical setting to the parents. | PowerPoint lectures, online video on DECIPHER | Role play. Trainers play the parents of the case assigned to each team. Team members act as clinicians and receive the parents at the consultation to return the information. They were supported by experienced genetic counsellors during this consultation. This is followed by a group discussion. | - Reference articles or online resources for the different genetic disorders diagnosed in the training cases. - PowerPoint lectures |
| Abbreviations: QF-PCR, Quantitative fluorescence- polymerase chain reaction; NGS, Next Generation Sequencing; SNV, Single Nucleotide Variation. | | | | |
